# EHMTI-0317. Transcranial direct current stimulation in chronic migraine: a pilot trial combining cathodal visual and anodal dlpfc stimulation

**DOI:** 10.1186/1129-2377-15-S1-G4

**Published:** 2014-09-18

**Authors:** R Baschi, SL Sava, V La Salvia, V De Pasqua, J Schoenen, D Magis

**Affiliations:** 1 Department of Neurology, CHR Citadelle, Liege, Belgium

## Background

Contrary to episodic migraine, chronic migraine (CM) is associated with sensitisation of visual cortex and depression that is characterized by left DLPFC hypoactivity. Transcranial direct current stimulation (tDCS) is able to activate (anode) or inhibit (cathode) the underlying cortex,thus of potential therapeutic interest in CM.

## Aim

To explore the effect in CM of simultaneous tDCS over the visual cortex and lDLPFC with a novel tDCS Cefaly° device.

## Method

We recruited 20 patients suffering from chronic migraine (n=15) or medication overuse headache (n=5)(ICHD-III beta 1.3 or 8.2).All had stable preventive treatment for at least 2 months. We applied anodal tDCS over F3 and cathodal over Oz: intensity 2 mA, duration 20min, daily for 8 weeks. Patients filled in headache diaries before (T0), during (T1) and after treatment (T2).We recorded CHEPS, QST, nBR and VEP at baseline (T0), immediately after (T1) and after 8 weeks (T2).

## Results

Total headache days decreased from 21.9/month at T0 to 15.7/month at T2 (-28.4%,p= 0.004).Severe migraine attacks were reduced by 43.7% (p= 0.05),headache hours by 30.2% (p= 0.02). The 50% responder rate for migraine days was 33.3%.There was no significant therapeutic effect in MOH patients.

tDCS had no effect on VEP and QST. It increased nBR habituation at T1 (p=0.05) but decreased habituation of frontal CHEPS at T2 (p=.04) in CM.

## Conclusion

These results suggests that tDCS inhibiting the visual cortex while activating lDLPFC has an interesting therapeutic potential in chronic migraineurs. A sham-controlled trial seems worthwhile.

No conflict of interest.

